# Supplementary material for: Programmable quantum emitter formation in silicon
Source: Nat Commun. 2024 May 27;15:4497. doi: 10.1038/s41467-024-48714-2 (PMC11130136; doi:10.1038/s41467-024-48714-2)
Supplement: Supplementary file 1 — Supplementary Information [file 41467_2024_48714_MOESM1_ESM.pdf]

Supplementary information for

Programmable quantum emitter formation in silicon

K. Jhuria[1, *], V. Ivanov[1,5], D. Polley[2, 6], Y. Zhiyenbayev[2], W. Liu[1], A. Persaud[1], W. Redjem[2, 7], W. Qarony[2], P. Parajuli[3], Q. Ji[1], A. J. Gonsalves[1], J. Bokor[2], L. Z. Tan[3], B. Kanté[2,4], and T. Schenkel[1,*]

[1]Accelerator Technology and Applied Physics Division, Lawrence Berkeley National Laboratory, Berkeley, CA 94720, USA

[2]Department of Electrical Engineering and Computer Sciences, University of California, Berkeley, CA 94720, USA

[3]Molecular Foundry, Lawrence Berkeley National Laboratory, Berkeley, CA 94720, USA

[4]Materials Sciences Division, Lawrence Berkeley National Laboratory, Berkeley, CA 94720, USA

[5]Virginia Tech National Security Institute, Blacksburg, VA 24060, USA

[6]College of Nanoscale Science and Engineering, SUNY Albany, Albany, NY, 12203, USA

[7]Department of Physics, BITS Pilani-Hyderabad Campus, Telangana 500078, India

(* t_schenkel@lbl.gov, kaushalya@lbl.gov)

1. Methods

Supplementary Figure 1 shows the depth profile of common elements in an as-received SOI wafer using secondary ion mass spectroscopy (SIMS). Residual hydrocarbons such as ^{12}C, H, and O are likely from ambient contamination during storage. Prior to ion implantation, the ^{13}C concentration is below the sensitivity limits of the SIMS measurement (~10^{16} $^{13}C/cm^2$). It can be noted that even in the as-received SOI wafer, there are already enough ^{12}C, H, and O atoms present to write common quantum emitters such as G centers (refer to Supplementary Note 9 for all-optical direct writing of W and G centers on as-received SOI sample without the need for any additional ion implantation or rapid thermal annealing).

SOI wafers were then implanted with carbon ions (^{13}C) by Kroko Implants. Prior to ion implantation, Stopping and Range of Ions in Matter (SRIM) simulations were performed to select implantation conditions for the desired C ion implantation depth in the Si device layer. We have targeted the implantation depth to be about in the center of the 220 nm thin Si device layer which is optimized for integrating the quantum emitters into photonic cavities [1,2]. As per the feedback received from the Stopping and Range of Ions in Matter (SRIM) simulation, ion energy of 38 keV at 7 degrees angle of incidence, provides a well-centered profile of C ions in the Si device layer.

These C-implanted substrates were then subjected to rapid thermal annealing under forming gas (H_2:10%, N_2:90%) ambiance at 800℃, for 120 seconds. The stepwise temperature profile for the annealing process representing the time for each step is shown in Supplementary Figure 1 b. We have verified the depth-resolved concentration profile for various common elements (i.e., ^{13}C, ^{12}C, H, O) in the pre-processed SOI wafer using SIMS as shown in Supplementary Figure 1 c.

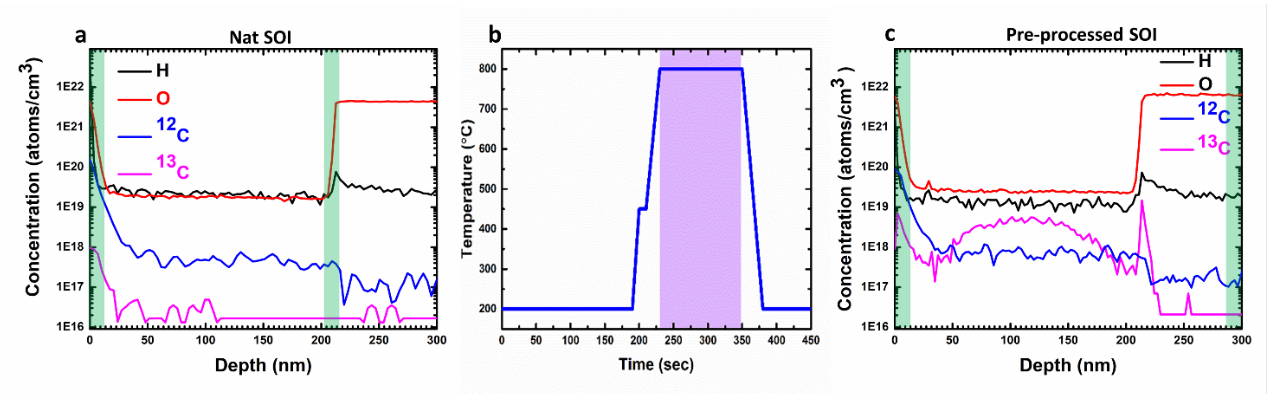

Supplementary Figure 1 SIMS depth profile of common elements in SOI device layers before and after ion implantation and rapid thermal annealing. a. SIMS profile representing the concentration of H, O, ^{12}C, ^{13}C in the as-received SOI. **b.** Annealing temperature as a function of time for rapid thermal annealing under forming gas ambiance (H_2:10%, N_2:90%) at 800 °C for 120 seconds to form pre-processed SOI samples. **c.** SIMS profile representing the concentration of H, O, ^{12}C, and ^{13}C in the pre-processed SOI. SIMS data near the surface and the silicon-SiO_2 box interface are highlighted in green and show transients to local changes of the relative ionization probabilities.

Supplementary Figure 1 c shows SIMS profiles for the pre-processed SOI. As simulated with SRIM, an ion energy of 38 keV of ^{13}C ion was used to target the center of the 220 nm thin Si device layer. Indeed, a nice ^{13}C profile centered around the desired depth was also observed with SIMS as shown in Supplementary Figure 1 c. The SIMS profile was measured after the rapid thermal annealing of ^{13}C implanted SOI.

We point out that the hydrogen profiles from SIMS before and after annealing under forming gas ambient look very similar. Before forming gas annealing we do not observe any C_i centers, after ion implantation and forming gas annealing, we do. An explanation is that hydrogen bonding configurations are redistributed during ion implantation and then during thermal annealing in forming gas, leading to the formation of C_i centers (as well as H and T centers, and the passivation

of G centers, depending on annealing times and temperatures, see Supplementary Note 7 below).

2. C_i center formation in SOI with ion implantation and thermal annealing under forming gas

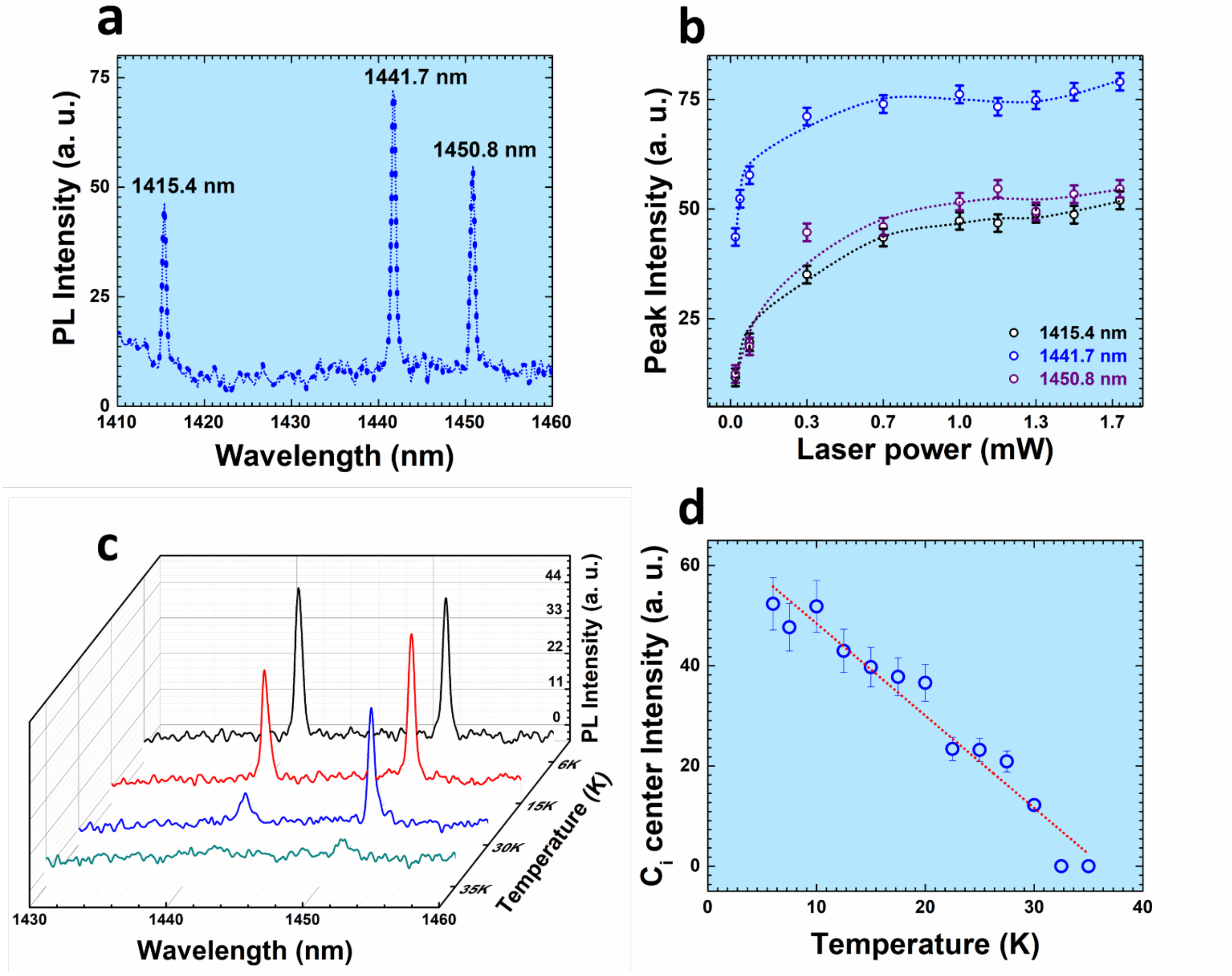

Supplementary Figure 2 Bright C_i center realization in SOI with C ion implantation followed by forming gas annealing. a. PL spectra obtained at 6 K with continuous 532 nm laser excitation representing the zero-phonon line (1415.4, 1441.7, 1450.8 nm) corresponding to the C_i center (these peaks can be associated with the different charged state of the C_i center). **b.** Peak amplitude variation as a function of excitation laser power shows a ~1:2 intensity ratio between the 1441.7 nm and the two other peaks (1415.4 and 1450.8 nm). **c-d.** Temperature response of the emission peak at ~1453 nm and the corresponding PL spectrum shows the robustness and

evolution in the C_i center emission from 6-37.5 K. Error bars shown in Supplementary Figure 2 b-d are statistical errors mapping to fluctuation in the peak amplitude in PL spectrum.

Supplementary Figure 2 a show the PL spectrum measured at 6 K, representing the ZPL corresponding to the C_i center. Here, the emission peaks at 1415.4 nm, 1441.7 nm, and 1450.8 nm are associated with different charge states of the C_i center containing ^{13}C that was introduced during implantation. A slight shift in the previously reported ZPL associated with the neutral state of the C_i center (i.e., 1448 nm) with that of the one obtained in this study at 1450.8 nm can be attributed to strain effects[3] and the presence of hydrogen in bright C_i centers. Supplementary Figure 2 b shows the dependence of the excitation power on the PL peak intensity of the ZPLs (1415.4, 1441.7, 1450.8 nm).

Depending on the measurement position on the sample, a distribution in the ZPLs was also observed (see Supplementary Note 3 for details on the statistics). The intensity variation follows a power function and saturates after a threshold excitation power of ~ 0.7 mW. A ratio of ~1:2 was observed between the intensities of the 1441.7 nm peak and other emission peaks, which is also dependent on the measurement location.

The temperature dependence of PL emission was also monitored to understand the stability and robustness of the emitter. Supplementary Figure 2 c shows the peak intensity of the C_i centers as a function of temperature (the measurement shown in Supplementary Figure 2 c was undertaken at a different location on the sample from the one shown in Supplementary Figure 2 b. A sublinear dependence between the PL peak intensity and the temperature was observed from 6-37.5 K, with PL intensity decreasing with temperature due to increased phononic and non-radiative contributions [4]. PL emission peak intensity corresponding to different temperatures are shown in Supplementary Figure 2 d. Similar measurements were performed during several temperature cycles and the emission from C_i centers was found to be unaffected reflecting their robustness and stability.

3. C_i center statistics on ZPL and high-resolution spectra for linewidth estimation

As discussed in the main manuscript, emission peaks corresponding to different charge states of the C_i center have ZPLs scattered within a few nm from the literature value related to the neutral state of the C_i center (i.e., 1448 nm). Supplementary Figure 3 a show the PL spectra representing different charge states associated with C_i centers measured at 20 different locations distributed in the area of ~5 mm^2 on the pre-processed SOI sample. Supplementary Figure 3 b shows an extremely narrow linewidth of ~0.03 nm measured with the highest grating (1200 g/mm, having a resolution of ~0.03 nm) indicating the actual linewidth to be even smaller than the one measured.

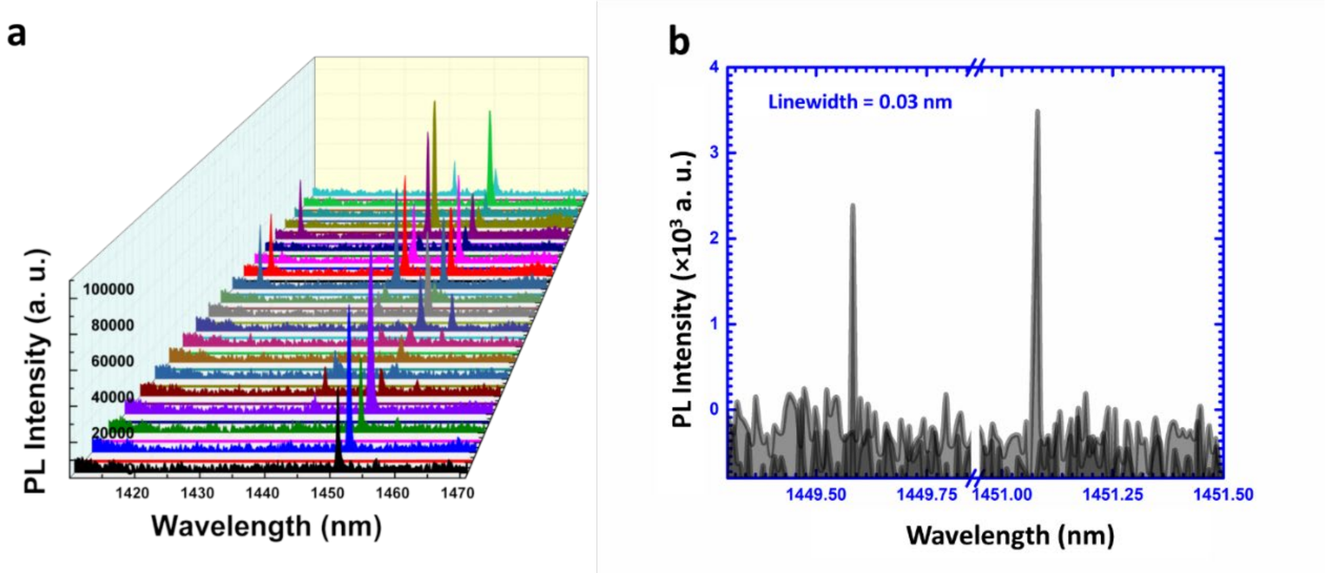

Supplementary Figure 3 Distribution in the zero-phonon lines (ZPLs) associated with different charge states of the C_i center and high-resolution PL spectra for linewidth estimation. a. PL spectra representing different charge states associated with C_i centers measured at 20 different locations distributed in the area of ~5 mm^2 on the pre-processed SOI. **b.** The High-resolution PL spectrum for C_i centers was measured with 1200 g/mm grating. Linewidth of ~0.03 nm is limited by the resolution of the spectrometer with the highest grating.

4. Fs laser irradiation for deterministic writing and erasing of G and C_i centers

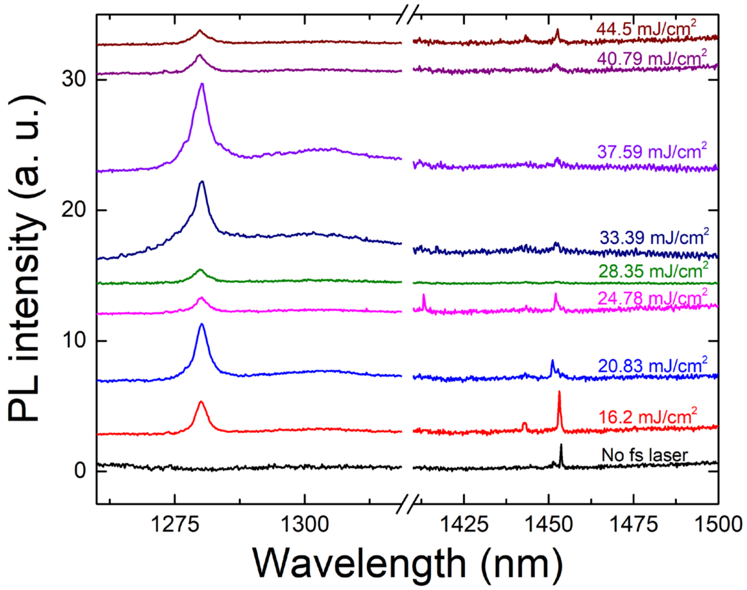

Supplementary Figure 4 PL measurements for G and C_i centers before and after fs irradiation in pre-processed SOI. a. PL spectra starting from no fs irradiation (showing only C_i center emission) to single pulse irradiation at varied fluences (showing the evolution in the G and C_i center emission resulting from fs laser driven writing and erasing).

As described in the manuscript, a single fs pulse irradiation with varied laser fluences was employed to write and erase various quantum emitters in particular G and C_i centers. Supplementary Figure 4 shows the PL spectra corresponding to the above-mentioned emitters after fs irradiation at different laser fluences. Here an evolution in the PL emission can be observed for both emitters. Supplementary Figure 4 shows the PL peak intensity from both emitters.

5. First-principles calculations

5.1. Charge state stability of C_i centers in silicon

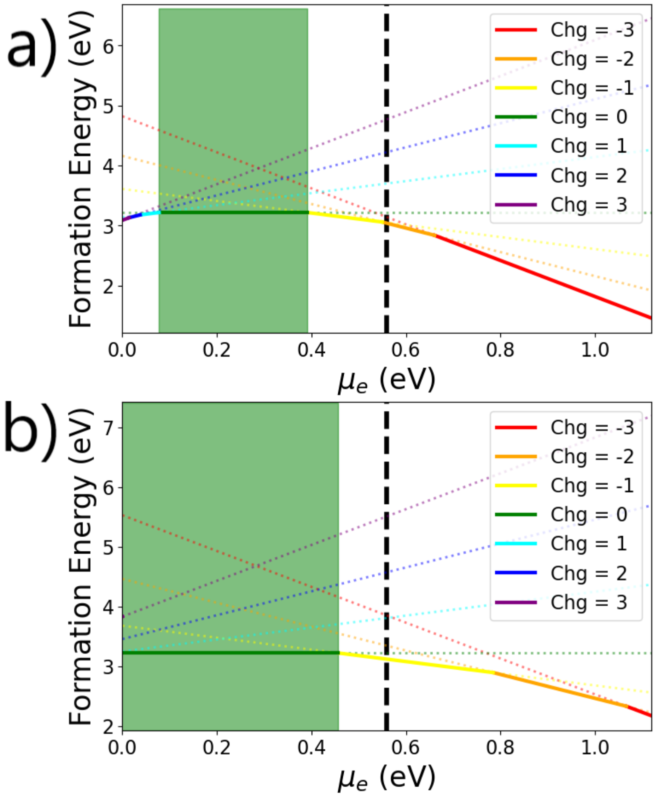

Supplementary Figure 5 Formation energy vs. Fermi level position in the silicon band gap for the C_i center. Formation energies for charge states ranging from -3 to +3 as a function of the Fermi level are plotted as dashed lines, with a solid line showing the most stable charge state at each level of doping. The black vertical line shows the position of the Fermi level for neutral, undoped silicon. The shaded green region denotes the region where the neutral charge state is the most stable. Results are shown without **(a),** and with **(b)** a finite size correction due to periodic boundary conditions.

The formation energies of various charge states of the C_i-center in silicon were computed as a function of the position of the Fermi level in the gap, using the Spinney package [5], with and without the finite size correction scheme of Kumagai and Oba[6] .

5.2 Hydrogen-modified structures of the G-center

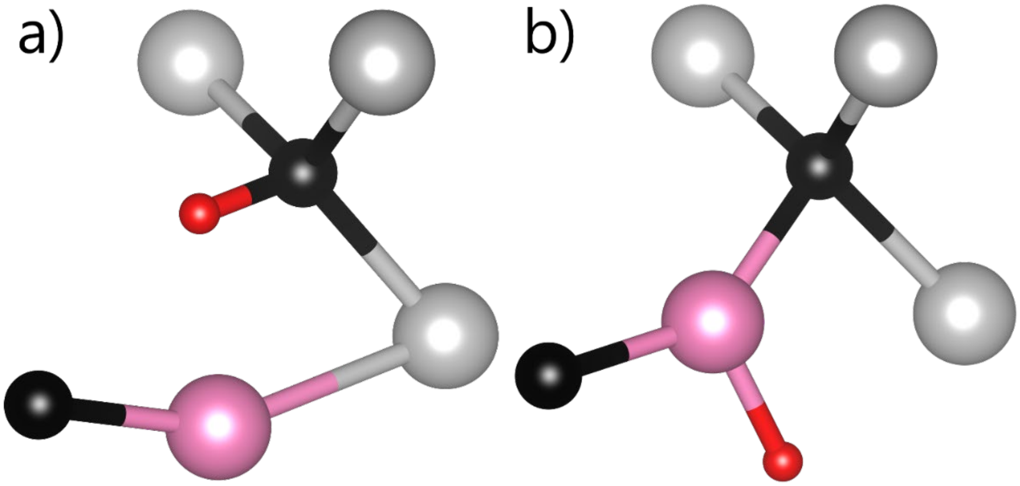

Supplementary Figure 6 Two structures of the G center B-form augmented by interstitial hydrogen. The possible structures of the G center Type-B modified by hydrogen are shown, with the hydrogen bonded to (a) the substitutional carbon, or **(b)** the interstitial silicon. Atoms are colored to denote silicon (gray), carbon (black), self-interstitial silicon (pink), and interstitial hydrogen (red).

5.3. Linewidth Broadening of the C_i center under strain

To understand the effect of local disorder, i.e. vacancies and self-interstitials in silicon, the C_i-center was embedded into a 3x3x3 silicon supercell in conjunction with a vacancy (216 possible sites) or interstitial (108 possible sites). These were down-selected to include only the structures where the separation between the C_i-center and the disorder (vacancy/interstitial) was greater than 0.8nm, which is more relevant for the experimental regime. The presence of a vacancy/interstitial in the supercell can serve as a trap for electrons or holes; consequently, the intrinsically stable charge state is used. Supplementary Figure 7 shows the resulting shift in zero phonon line of the C_i-center as a function of distance to a vacancy/interstitial in each structure, while Supplementary Figure 7 b,c, show histograms of the resulting shifts. Overall, the broadening of the C_i-center appears slightly narrower than that of the G-center [3], indicating a

lower sensitivity to local disorder.

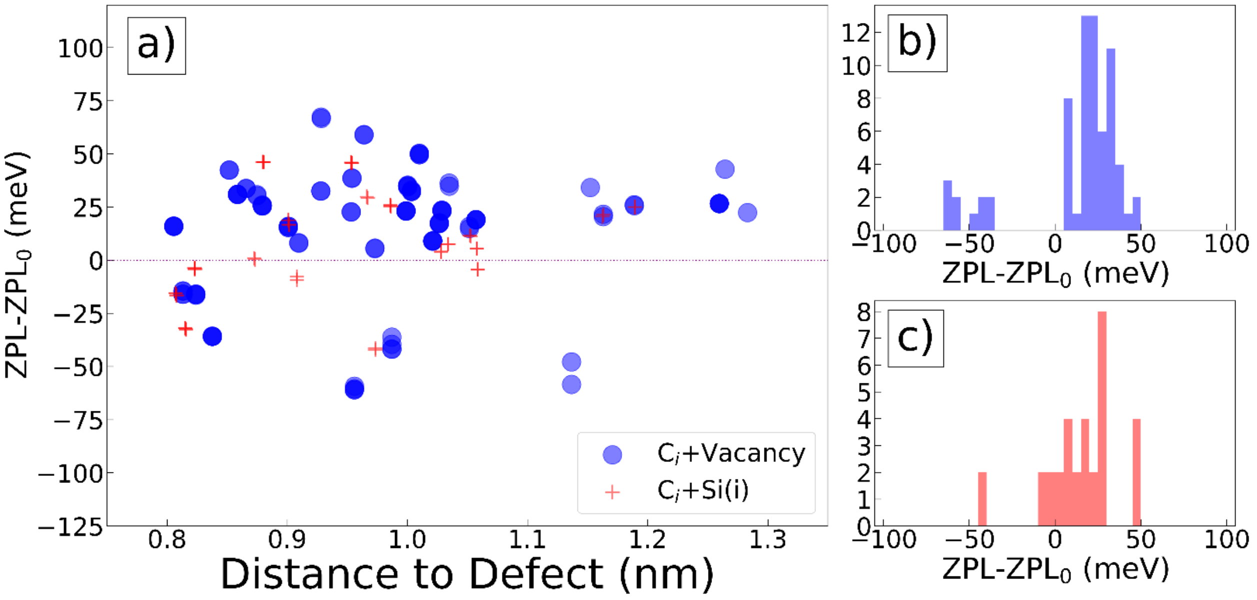

Supplementary Figure 7 Scatter plot (a) of zero phonon line shifts of the C_i-center as a function of distance to disorder, vacancies (blue) and silicon self-interstitials (red). Histograms of the zero phonon line distributions for vacancies **(b)** and interstitials **(c)**

6. Isolated G and C_i center formation post fs laser annealing

6.1 Statistics on single center formation post fs laser irradiation

In Fig 3, the observation of $g^2(0)$ <0.5, provides compelling evidence for single photon emission originating from the G and C_i centers following fs laser irradiation at ~8 mJ/cm² fluence. To expedite our analysis, we conducted additional optical characterizations, including photoluminescence (PL) spectra, and time-resolved PL (TR-PL) to obtain optical lifetimes and polarization sensitivity PL emission (see Supplementary Figure 9 a-c). Notably, laser spots irradiated within the 8-12 mJ/cm² fluence range exhibited extended lifetimes and higher polarization sensitivity. Subsequent second-order correlation measurements on two such spots for each center (G and C_i) consistently revealed $g^2(0)$ <0.5. Supplementary Figure 9 illustrates the optical characterization outcomes of multiple isolated laser spots post fs laser irradiation, providing insights into potential single-center identifications with near-unity probability.

Angle-dependent photoluminescence for the C_i center was calculated from DFT transition dipole matrix elements. The polarization direction (v) was rotated in the xy plane, with the z-direction

being along the C-Si bond. The C_i center is not emissive for polarization in the z-direction. Photoluminescence intensity was taken to be proportional to $|<d \, . \, v>|^2$, with d being the transition dipole (Supplementary Figure 8).

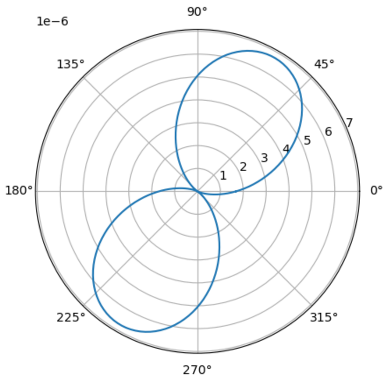

Supplementary Figure 8 Angle dependent PL for Isolated C_i center calculated from DFT transition dipole matric elements.

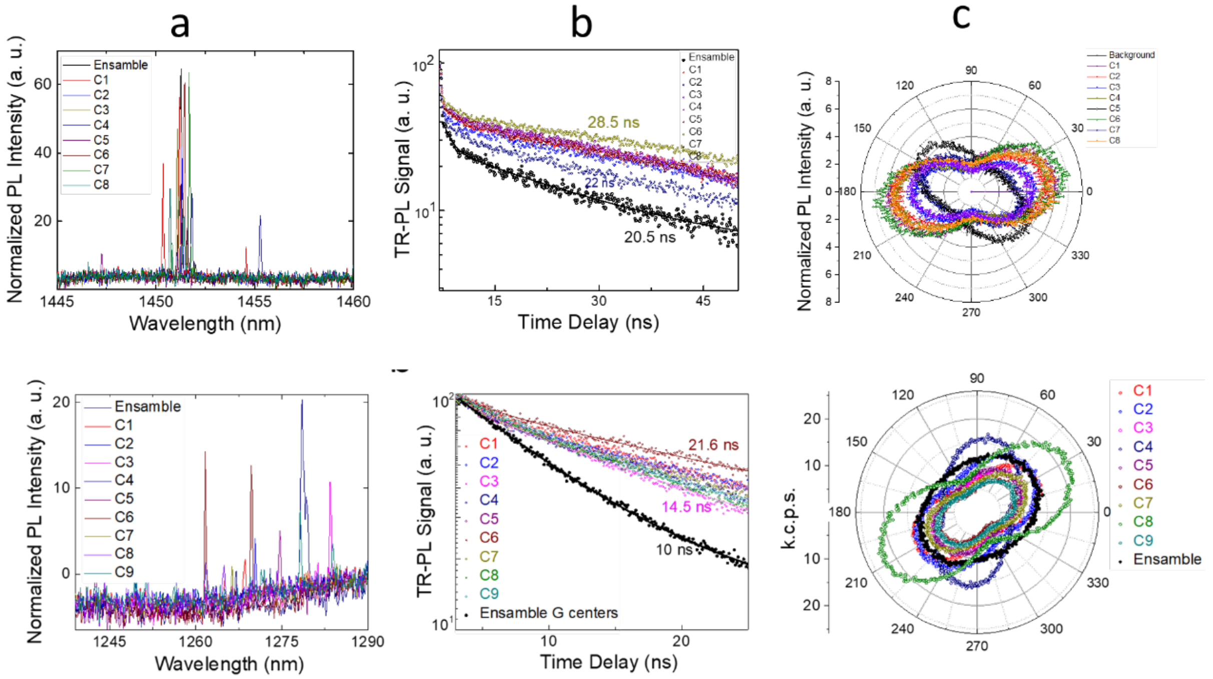

Supplementary Figure 9 Systematics on the optical properties of ~ 10 isolated C_i and G centers (top and bottom row respectively) post fs irradiation in the range of 8-12 mJ/cm^2 laser fluence. **a.** PL Spectra corresponds to an ensemble and different isolated G and C_i centers. **b.** TR-PL signal corresponds to an ensemble and different isolated G and C_i centers along with extracted optical

lifetime range obtained after first-order decay function fit**. c.** Polarization sensitivity PL from an ensemble and isolated G and C_i centers.

6.2 Background subtraction post-second-order correlation measurement

Background correction was performed to suppress impact of dark counts from detectors and leaked photons (from laser and ambient). The background corrected signal ($g^2(\tau)_{\text{background corrected}}$) was obtained as below:

$$g^2(\tau)_{\text{background corrected}} = \left[\frac{g2(\tau) - (1-\rho 2)}{\rho 2}\right] \text{..........................} (1)$$

Here ρ is the signal to background ratio[7].

6.3 Stability of the C_i center under laser excitation

We monitored the PL emission signal from the C_i center formed after fs laser irradiation continuously for 30 mins and no significant change in the emission signal was observed.

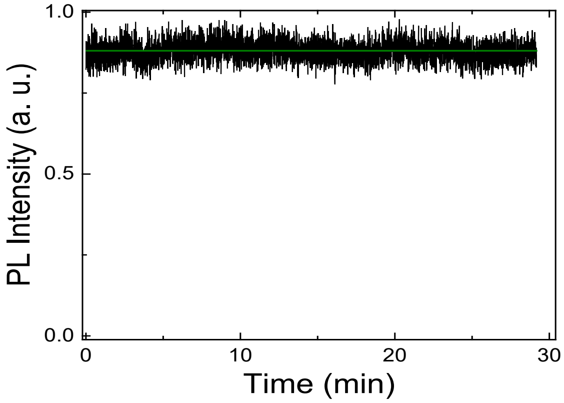

Supplementary Figure 10 Long-time stability of C_i center under 532 nm laser excitation.

7. Forming gas annealing parameters for the programmable formation of quantum emitters

We have explored a large range of forming gas annealing parameters in order to selectively form a series of potential spin-photon interface candidates while passivating the more common G centers. Supplementary Figure 11 a-b shows the PL spectra for a selective telecom band range to show the passivation of common G center and forming promising Spin-photon interface candidates such as T and the H-centers respectively. While the T centers were formed after annealing at 1000 °C for 60 seconds, H centers were observed after 20 seconds. Both T, H centers involve C and H atoms in their structure, similar to C_i centers (as detailed in the main manuscript)

[8].

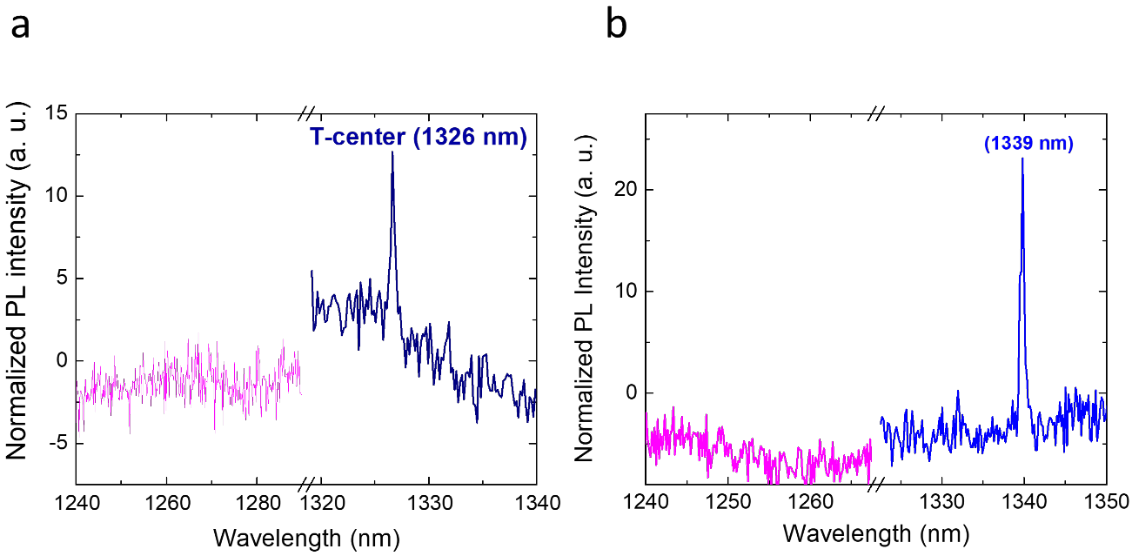

Supplementary Figure 11 Forming gas annealing time-dependent selective formation and passivation of T and H centers while passivating the common G center. a. PL spectra corresponding to T centers after rapid thermal annealing of C implanted SOI at 1000 °C for 60 seconds. **b.** H center PL spectra after the annealing at 1000 °C for 20 seconds.

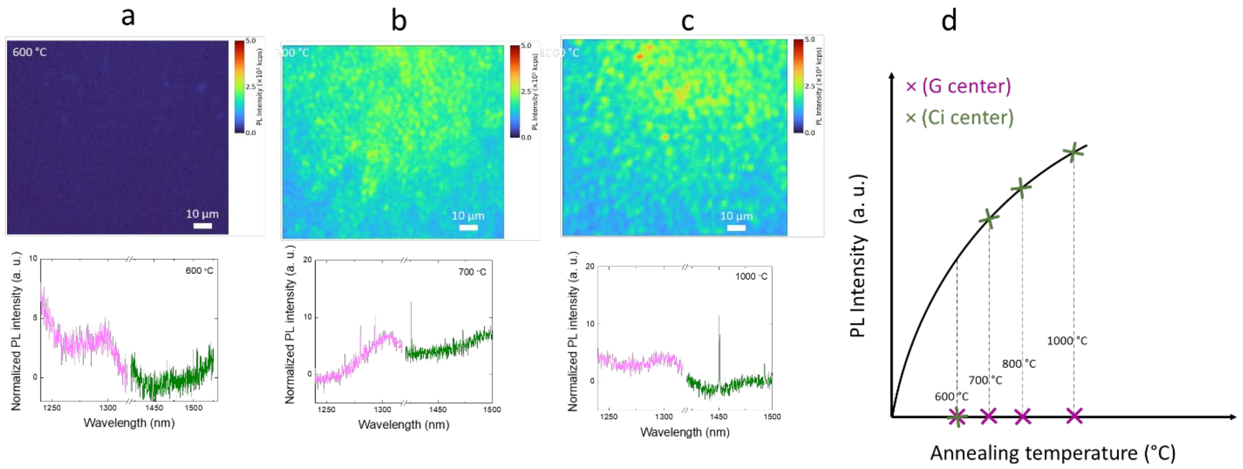

Supplementary Figure 12 Forming gas annealing temperature-dependent selective formation and passivation of G and C_i centers. a-c. Photoluminescence raster scan (top row) and spectra (bottom raw) in the telecom band range after annealing at 600, 700, and 1000 °C respectively at a constant annealing time of 120 seconds

Similarly, as mentioned in the main manuscript, C_i centers can also be observed for several annealing parameters. To assess annealing temperature dependence, samples from 600, 700, 800, and 1000 °C were tested, maintaining a constant annealing time of 120 seconds were studied.

No color centers were observed at 600 °C, potentially attributed to lattice healing after ion implantation. Both G and C_i centers were detected at 700 °C, while only C_i centers were observed in the 800-1000 °C range (see Supplementary Figure 12 a-d). In the latter case, with a constant annealing temperature of 800 °C, varying the annealing time (20, 60, 80, 100, and 120 seconds) revealed consistent trends in color center formation. Notably, shorter annealing times exhibited a window for single G and C_i centers (see Supplementary Figure 13 a-d). This controlled and selective formation of different quantum emitters maps to the programmability aspect adjusting different annealing parameters.

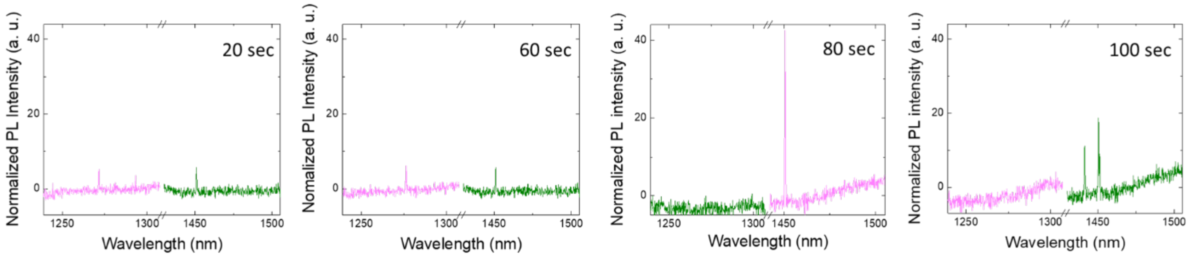

Supplementary Figure 13 Forming gas annealing time-dependent selective formation and passivation of G and C_i centers. a-d. PL spectra correspond to different annealing times starting from 20, 60, 80, and 100 seconds while keeping the annealing temperature constant.

8. Domination of ensemble G centers above Si melting threshold fs laser irradiation

For ensemble G centers, we extended our analysis to encompass laser fluences ranging from the 10s to 1000s of mJ/cm². Our observations reveal a substantial broadening in the linewidth, contingent upon the irradiation conditions, reflecting the silicon lattice conditions post-irradiation. Under below-melting threshold conditions, the ensemble linewidth widens, as illustrated in Fig 2a. Conversely, surpassing the melting threshold, where lattice recrystallization occurs, and maintaining the same laser fluence as indicated in the aforementioned reference, results in a narrower linewidth. This narrowing aligns with the healed silicon lattice post-recrystallization at significantly higher fluences. For a precise comparison, we plotted the G center spectra at higher laser fluences and noted a linewidth of 1 ± 0.1 nm for the higher laser fluence range. Importantly, this value remains comparable to the state-of-the-art linewidth for ensemble G centers. Supplementary Figure 14a and b show the PL spectra and linewidth of the ensemble G

center post above Si melting threshold fs laser irradiation respectively. For the given range, the minimum linewidth of ~ 1nm was observed for the ensemble G center ~ 2400 mJ/cm^2 .

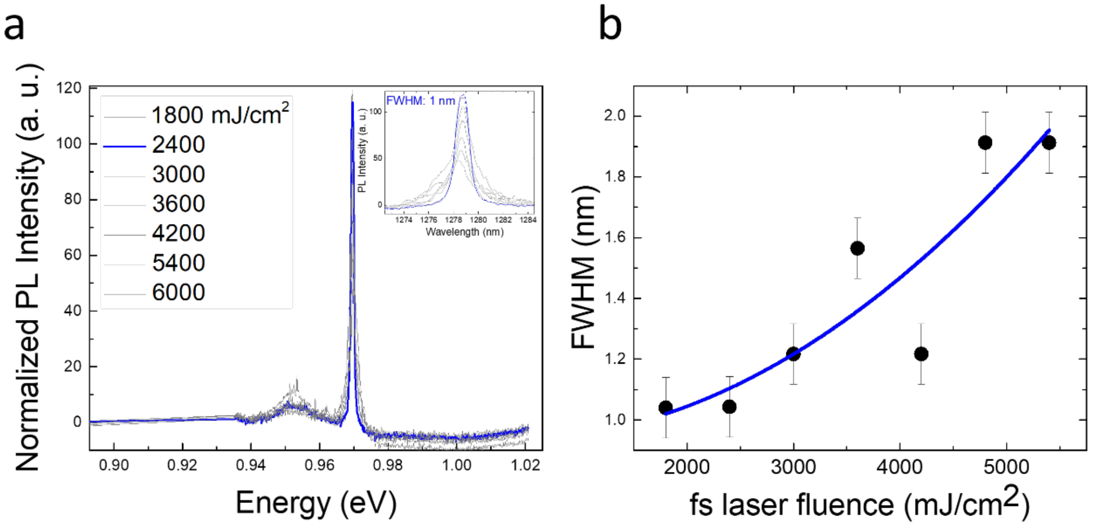

Supplementary Figure 14 G center ensemble dominance above Si melting threshold. a. PL spectra from the G center ensemble after above Si melting threshold irradiation with single fs laser pulses of varied fluences. **b.** G center linewidth dependence on the fs laser fluence. Errorbars are statistical errors from sample to sample that map to variations in the pulse energy of the fs laser output observed in our experiments.

9. All-optical writing and erasing of W and G centers in SOI

While the G and C_i centers were programmed via fs laser pulse irradiation in pre-processed SOI, direct writing and erasing of G centers were also established in as-received SOI without any external ion implantation and thermal annealing. We repeated a similar single fs laser pulse irradiation on the as-received SOI and checked the emission spectra in the telecom band range. Precise control over the G centers was observed in the lower laser fluence regime followed by W centers writing at the near damage threshold fluence range.

Supplementary Figure 15a shows the PL spectrum corresponding to G centers ZPL and phonon sidebands after a single fs pulse irradiation at a laser fluence of 28.35 mJ/cm^2. The observed trends in the PL peak intensity around this fluence are shown in Supplementary Figure 15b. It is clear from the trend that the density of G centers can be precisely controlled by varying the laser fluence.

A recent study using FIB to form G centers demonstrated the necessity of C pre-implantation, without which subsequent processing with a Si-focused ion beam would lead to the formation of only W centers. Our current work with fs laser pulses provides evidence for direct writing and precise control over the density of G center formation on the as-received SOI sample[9].

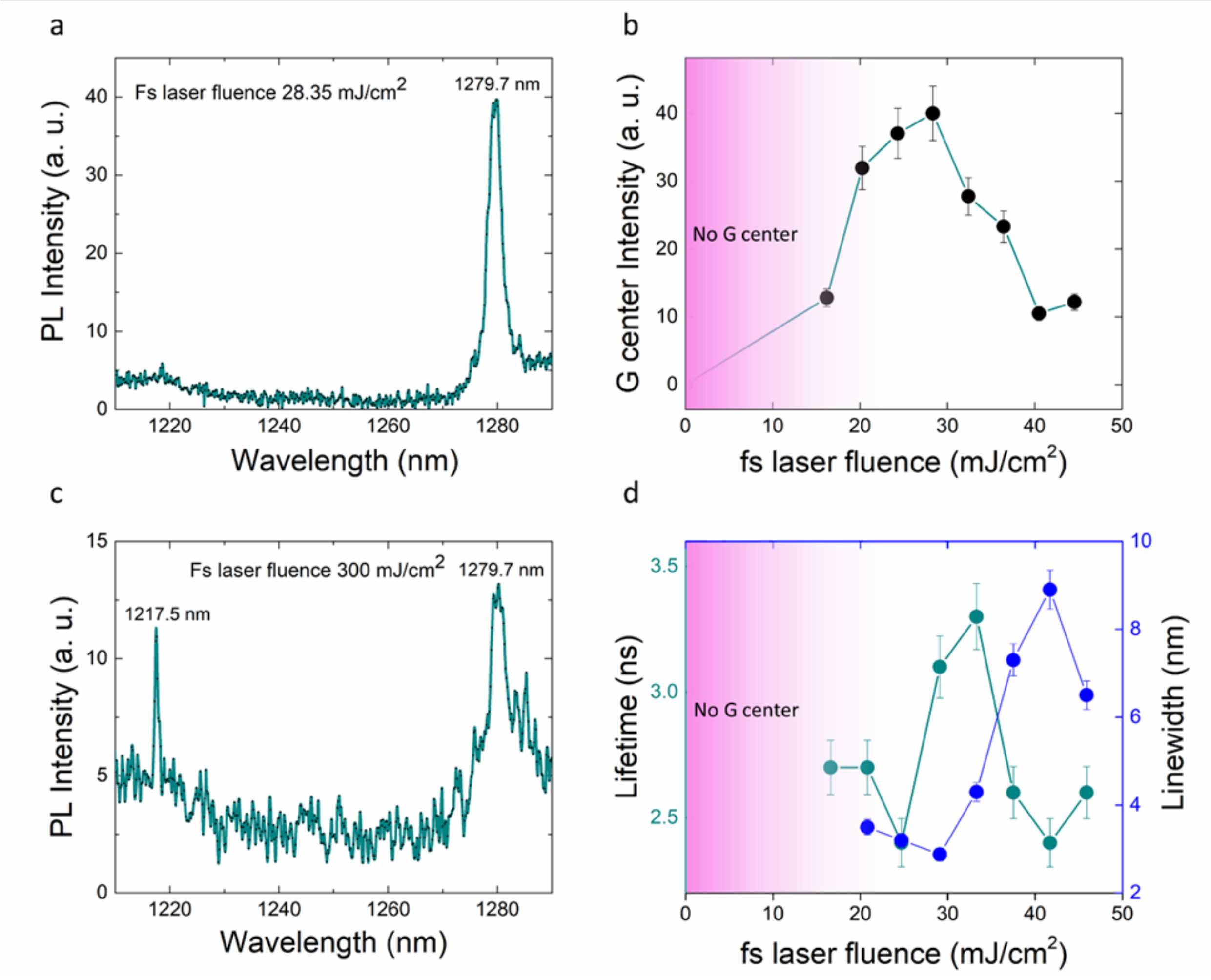

Supplementary Figure 15 All-optical writing and erasing of G centers with direct fs laser pulse irradiation on as-received SOI. a. PL response from G centers showing the ZPL and the corresponding phonon side band formed in as-received SOI substrate after direct irradiation with a single fs laser pulse of 28.35 mJ/cm^2 fluence. **b.** The peak amplitude of the PL spectra from G centers as a function of fs laser fluence per pulse represents the writing and partial erasing of these emitters with fine control over the density. **c.** PL spectra after fs irradiation with near damage threshold fluence of ~300 mJ/cm^2 leading to the formation of radiation damage related W centers along with G centers. **d.** The extracted lifetime of G centers after fitting the TR-PL signal with a single decay function along with their linewidths after fs irradiation for a series of laser fluences. Errorbars in Supplementary Figure 15 b-d are statistical errors from sample to sample that map to variations in the pulse energy of the fs laser output observed in our experiments

Additionally, no W centers were observed while working with fs laser pulses in the lower fluence range, further suggesting that this approach is superior for maintaining the integrity of the Si lattice. Supplementary Figure 15c shows the PL spectra after the as-received SOI was irradiated with near Si damage threshold fs laser pulse fluence of ~300 mJ/cm^2. W centers were formed near these high fluences consistent with their structure consisting of three silicon interstitials that are formed by damaging the silicon lattice. We performed TR-PL measurements to extract the non-radiative lifetime and to gain insights into the defect dynamics involved in emitter formation and the resulting quality of G centers. Supplementary Figure 15d shows the non-radiative lifetime and linewidth of G centers after fs irradiation at different laser fluences. An inverse trend for lifetime and linewidth was observed as a function of fs laser fluence.

Supplementary References

1. MacQuarrie, E. R. *et al.* Generating T centres in photonic silicon-on-insulator material by ion implantation. *New J. Phys.* **23**, 103008 (2021).
2. Prabhu, M. *et al.* Individually addressable and spectrally programmable artificial atoms in silicon photonics. *Nat. Commun.* **14**, 1–7 (2023).
3. Liu, W. *et al.* Quantum Emitter Formation Dynamics and Probing of Radiation-Induced Atomic Disorder in Silicon. *Phys. Rev. Appl.* **20**, (2023).
4. Beaufils, C. *et al.* Optical properties of an ensemble of G-centers in silicon. *Phys. Rev. B* **97**, 035303 (2018).
5. Arrigoni, M. & Madsen, G. K. H. Spinney: Post-processing of first-principles calculations of point defects in semiconductors with Python. *Comput. Phys. Commun.* **264**, 107946 (2021).
6. Kumagai, Y. & Oba, F. Electrostatics-based finite-size corrections for first-principles point defect calculations. *Phys. Rev. B - Condens. Matter Mater. Phys.* **89**, (2014).
7. Huang, J. *et al.* Exciton-polariton dynamics of the single site-controlled quantum dot-nanocavity in the coexisting strong-weak coupling regime. *New J. Phys.* **25**, (2023).
8. Davies, G. *The Optical Properties of Luminescence Centers in Silicon*. (North-Holland, 1989).
9. Quard, H. *et al.* Femtosecond laser induced creation of G and W-centers in silicon-on-insulator substrates. (2023).
